# *Plasmodium* Actin‐Like Proteins 3 and 5a Are Required for Subsequent Steps of Mosquito Infection

**DOI:** 10.1111/mmi.70108

**Published:** 2026-08-21

**Authors:** Caroline Busse, Yukino Kobayashi, Andrea Diers, Annika M. Binder, Friedrich Frischknecht, Ross G. Douglas

**Affiliations:** ^1^ Institute of Veterinary Physiology and Biochemistry Justus Liebig University Giessen Giessen Germany; ^2^ Integrative Parasitology, Centre for Integrative Infectious Disease Research Heidelberg University Medical School Heidelberg Germany; ^3^ German Center for Infection Research DZIF, Partner Site Heidelberg Heidelberg Germany; ^4^ Excellence Cluster SynthImmune Heidelberg University Heidelberg Germany

**Keywords:** actin‐like protein, actin‐related protein, mosquito transmission, ookinete, *Plasmodium*

## Abstract

Actin superfamily members are critical for the biology of eukaryotes and archaea. Actin‐related proteins (Arps) are a subgroup within the actin superfamily and play essential roles in trafficking, replication and motility. The genome of the malaria parasite *Plasmodium* contains a set of Arps unique to apicomplexans, termed actin‐like proteins (Alps). However, the importance and specific roles of many of these Alps in *Plasmodium* progression are not yet understood. Here, we determined the functional contribution of *Plasmodium berghei* Alp3 and Alp5a (recently relabelled as Arp3) by generation of knock‐out (KO) lines and their subsequent characterisation across different life cycle stages. Deletion of either Alp did not affect blood stage growth, gametogenesis and ookinete gliding motility. However, deletion of Alp5a led to smaller and fewer oocysts as well as severely impaired sporozoite formation. The Alp3KO line had highly reduced oocyst loads compared to wild‐type parasites. This striking decrease was due to impaired ookinete penetration of the mosquito midgut epithelium. Our study shows that both Alp3 and Alp5a are indispensable for *Plasmodium* transmission at different steps of initial mosquito infection, provides insights into the role of specific unique members of the actin superfamily during parasite progression and the requirements for efficient midgut penetration.

## Graphical abstract

Actin‐like proteins (Alps) are highly conserved, apicomplexan‐specific members of the actin superfamily. Using reverse genetics, we have identified that Alp3 and Alp5a are required at separate steps of transmission of the malaria parasite to its mosquito host. Alp3 is critical for ookinete penetration of the mosquito midgut, while Alp5a is important for oocyst development and sporozoite formation.

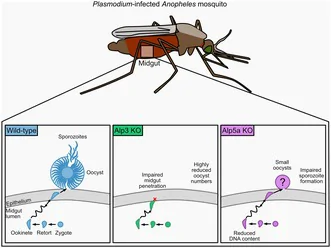

## 1 Introduction

Malaria is a deadly tropical disease caused by parasites of the genus *Plasmodium* (World Health Organization 2025). During their life cycle, the parasites need to infect and adapt to very different host environments. This requires active motility as well as transcriptional, proteomic, metabolic and morphological changes in order to facilitate effective parasite replication and spread (Douglas et al. 2015; Cowman et al. 2016). After a blood meal, gametocytes transform into male and female gametes in the mosquito bolus that fuse to form a zygote which subsequently develops into an ookinete (Angrisano et al. 2012; Darif et al. 2025). Ookinetes actively move by gliding motility and penetrate the mosquito's midgut epithelium where they transform into oocysts, which mature at the basal lamina and produce hundreds of sporozoites per oocyst (Baton and Ranford‐Cartwright 2005; Douglas et al. 2015; Singer and Frischknecht 2023). These sporozoite egress into the haemocoel and then passively float through the haemolymph until invasion of the salivary glands for onward transmission (Douglas et al. 2015; Singer and Frischknecht 2023). Infection of the mosquito midgut represents one of the largest bottlenecks in the *Plasmodium* life cycle. Due to difficulties in culturing oocysts in vitro, and the relatively rapid nature of the infection process, little is known about this crucial mosquito stage and thus the proteins and protein dynamics required for these steps are still poorly understood (Smith and Barillas‐Mury 2016; Hentzschel and Frischknecht 2022; Bailey et al. 2026).

The actin superfamily consists of multiple members, including conventional actin and actin‐related proteins (Arps) (Kabsch and Holmes 1995; Hurley 1996). Arps are highly sequence divergent in comparison to conventional actin and are involved in processes such as intracellular trafficking, actin dynamics regulation and chromatin remodelling (Boyer and Peterson 2000; Oma and Harata 2011). Interestingly, *Plasmodium* has a reduced set of assigned classical Arp homologues (Gordon and Sibley 2005). The *Plasmodium* genome nonetheless contains a subgroup of six Arps found only in Apicomplexa, known as actin‐like proteins (Alps), which are highly sequence divergent from the classical Arps (Gordon and Sibley 2005). Recently two Alps (Alp5a and Alp5b) were found to be part of an unusual Arp2/3 complex (Hentzschel et al. 2025). In general, very little is known about Alp function in apicomplexan biology. Here, we investigated the roles of two particular Alps, Alp3 and Alp5a (Arp3), in the biology of *Plasmodium*.

As members of the actin superfamily, Alp3 and Alp5a are predicted to share the conserved actin‐fold core (Figure S1A). However, both Alps are significantly larger than conventional *Plasmodium* actin (539 and 574 amino acid residues for Alp3 and Alp5a respectively, compared to 376 residues for actin 1) (Figure S1A) and contain large insertions, many of which are located on the periphery. In terms of amino acid sequence, these Alps are highly divergent from *Plasmodium* actin 1 (13% identity and 28% similarity for Alp3 as well as 14% identity and 25% similarity for Alp5a) (Figure S1B,C). Through reverse genetics and imaging approaches, we identified key roles of these two Alps in ookinete penetration of the midgut epithelium and oocyst development indicating that these Alps, while likely operating independently from each other, are required for two subsequent steps of mosquito infection.

## 2 Results

### 2.1 Alp3 and Alp5a Are Not Required for Asexual Blood‐Stage Growth, Male Gametogenesis and Ookinete Gliding Motility

To investigate the roles of Alp3 and Alp5a in vertebrate and mosquito hosts, knock‐out (KO) lines were generated in the rodent model *Plasmodium berghei*. Linearised knock‐out vectors were transfected into schizonts (Figure S2). During limiting dilution, asexual blood‐stage growth of these knock‐out parasites was investigated. Relative growth rates were comparable to the complementation control, indicating that deletion of these Alps did not lead to strong impacts on blood‐stage parasites (Figure 1A). These knock‐out lines were then used to investigate the Alps' role in the mosquito host.

**FIGURE 1:**
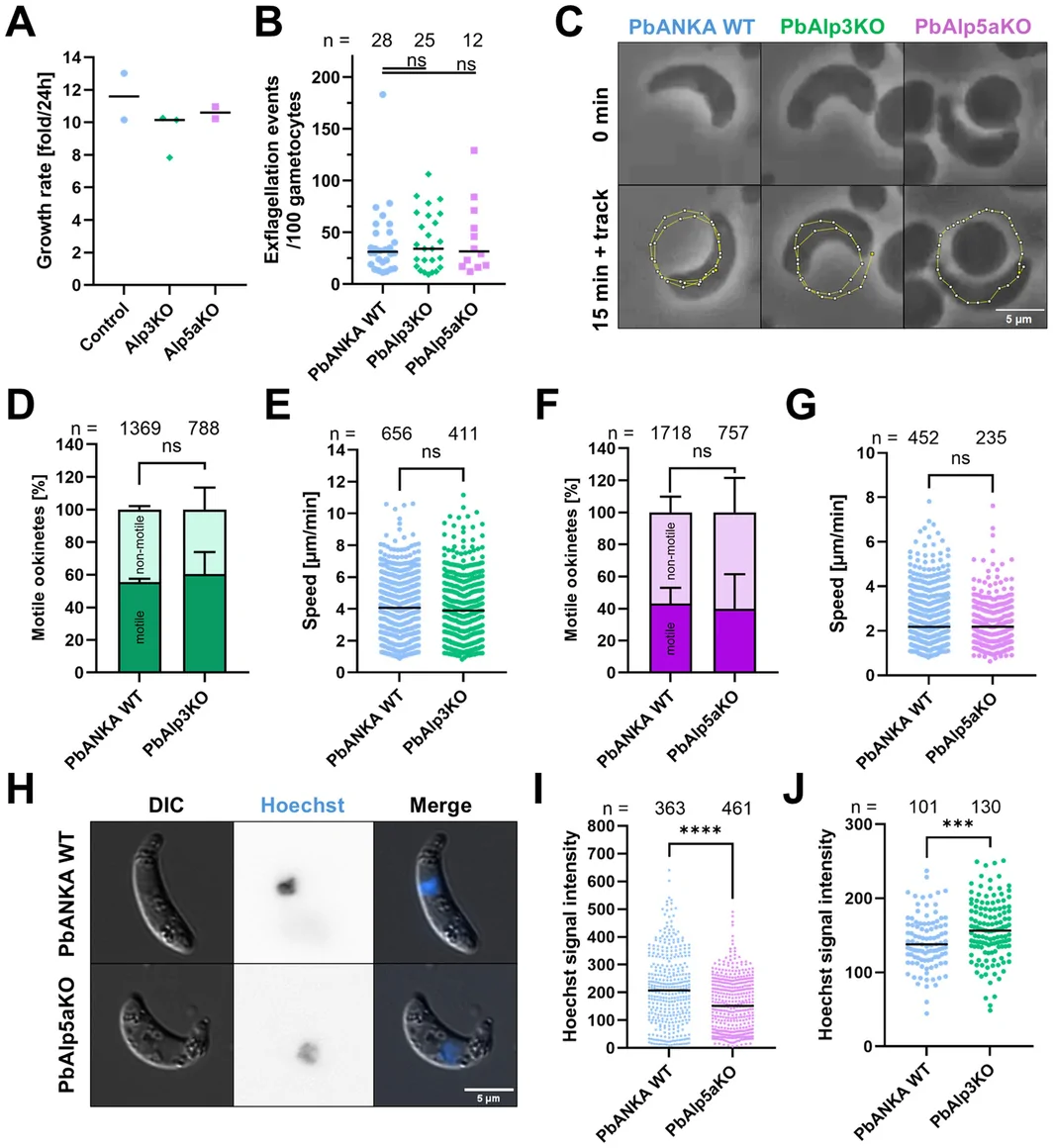
Alp3 and Alp5a are not required for blood stage growth, exflagellation or ookinete motility. (A) Knock‐out (KO) of *P. berghei* Alp3 and Alp5a does not affect asexual blood‐stage growth compared to control parasites. (B) Knock‐out of Alp3 and Alp5a does not affect exflagellation of male gametocytes compared to PbANKA wild‐type (WT). (C) Alp3KO and Alp5aKO ookinetes move in a circular pattern. Movement was tracked over 15 min. (D, E) Motile population and gliding speed of Alp3KO ookinetes are comparable to wild‐type. (F, G) Motile population and gliding speed of Alp5aKO ookinetes are comparable to wild‐type. (H) Hoechst signal is reduced in Alp5aKO ookinetes. (I) Alp5aKO ookinete nuclear Hoechst signal intensity is reduced by 25%. (J) Ookinete Hoechst signal intensity is not reduced in the Alp3KO line compared to wild‐type, indicating no essential role at this stage of the life cycle. Statistics were performed using Fisher's exact test (D, F) or Mann–Whitney test (E, G, I, J). ns, not significant; ***p < 0.001; ****p < 0.0001 (Table S1). In Figure 1A, growth rates were determined for individual clones in separate mice as indicated. For all mosquito stage experiments, at least two biological replicates were performed per parasite line.

To assess mosquito infection ability, we first investigated the Alps' role in exflagellation of male gametocytes. Compared to wild‐type parasites, no significant change in the number of exflagellation events was observed in Alp3KO and Alp5aKO parasites, highlighting the lack of either Alp3 or Alp5a involvement in this process (Figure 1B). Ookinetes are highly motile parasite stages that employ active gliding motility to traverse the mosquito's midgut epithelium to colonise a mosquito host. We thus explored the in vitro gliding motility of Alp3KO and Alp5aKO lines. Gliding motility of both knock‐out ookinete lines were comparable to wild‐type in terms of percentage of motile ookinetes and speeds (Figure 1C–G). Therefore, Alp3 and Alp5a do not play a major role in 2D in vitro ookinete gliding motility. Alp5a was recently identified as a member of an atypical Arp2/3 complex and knock‐out of fellow complex members ARPC1 and Alp5b (Arp2) lead to a 33% reduction of DNA content in ookinetes compared to wild‐type parasites (Hentzschel et al. 2025). To investigate the influence of Alp3 and Alp5a on the DNA content of ookinetes, Hoechst staining intensity was analysed. Nuclear staining intensity of Alp5aKO ookinetes was reduced by about 25% compared to wild‐type (Figure 1H,I), consistent with deletion of other complex members and a previous Alp5a knock‐out (Hentzschel et al. 2025; Varshney et al. 2025). In contrast, knock‐out of Alp3 did not negatively affect ookinete Hoechst signal intensity (Figure 1J).

### 2.2 Alp3 and Alp5a Are Required for Oocyst Formation

Knock‐out parasites were next investigated for their role in oocyst formation. Oocysts are sessile parasite midgut colonies which develop over the course of several days. Knock‐out parasite lines were fed to *Anopheles stephensi* mosquitoes and infection rates analysed (Figure 2A). Alp3 and Alp5a knock‐out lines were able to colonise mosquito midguts (Figure 2B) and the percentage of infected mosquitoes was comparable to the wild‐type control (Figure 2C). However, Alp3KO‐infected mosquitoes had approximately 99% reduced oocyst numbers (Figure 2D) and, in the case of the Alp5aKO line, total oocyst numbers were decreased by about 75% on days 11–12 after blood meal (Figure 2D). While the size of the few observed oocysts in the Alp3KO line was not negatively impacted, Alp5aKO resulted in reduced oocyst size by approximately 84% (Figure 2E). We generated complementation lines to investigate whether these reductions in oocyst numbers were caused by single‐gene knock‐outs (Figure S3). In both cases, oocyst numbers, and in case of the smaller oocysts for the Alp5aKO line, oocyst size was rescued in the complementation line (Figure 2D,E), indicating that Alp3 and Alp5a specifically are critical for oocyst formation.

**FIGURE 2:**
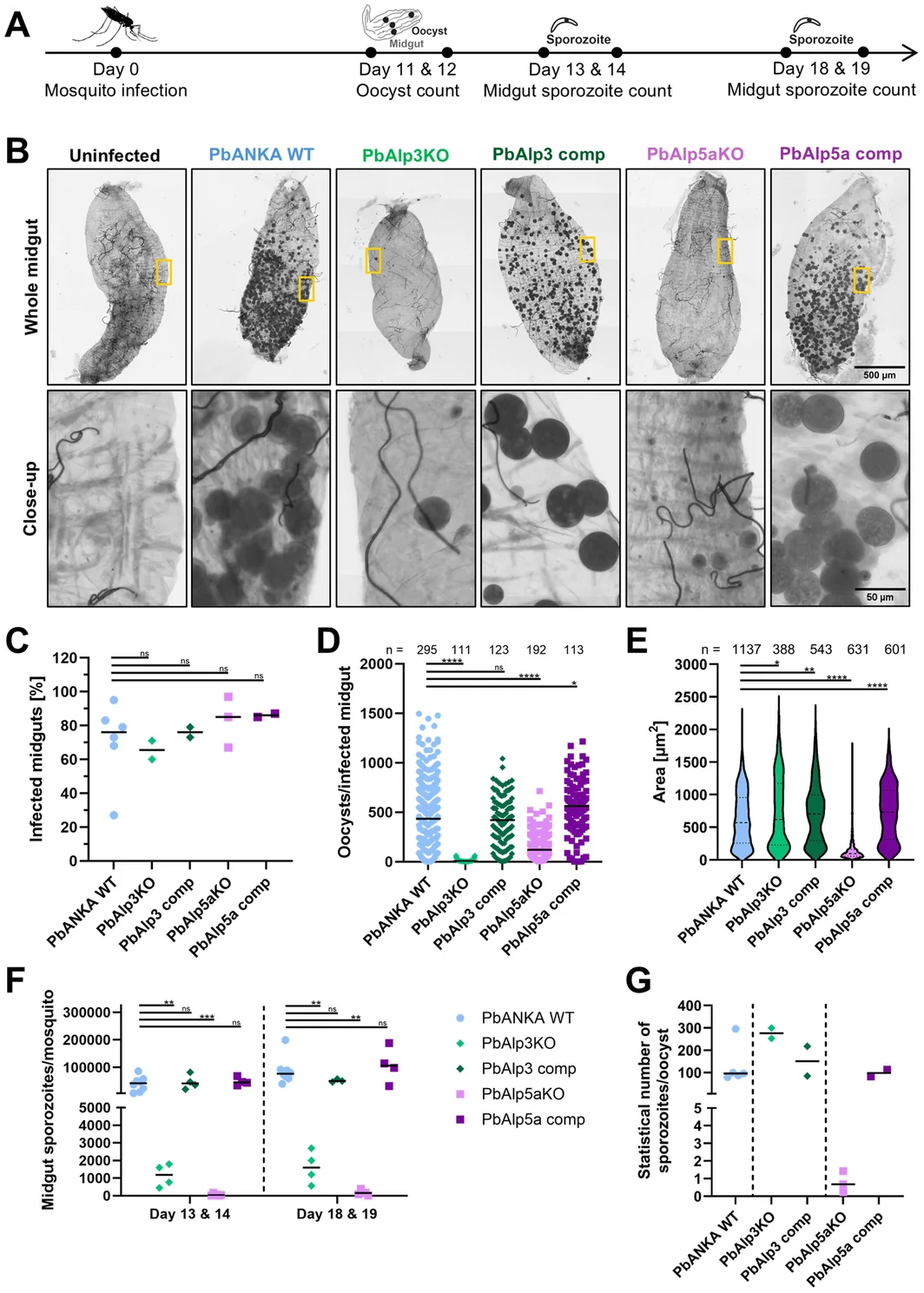
Alp3 and Alp5a are required for oocyst formation. (A) Scheme of mosquito analysis timeline. (B) Microscopy images of *Anopheles* midguts stained with mercurochrome. (C) Knock‐out of Alp3 and Alp5a did not affect infection rates of mosquito midguts. (D) Oocyst numbers in *Anopheles* midguts are reduced in the Alp3KO and Alp5aKO lines. (E) While knock‐out of Alp3 had no negative effect on oocyst size, Alp5aKO oocysts were much smaller than wild‐type. (F) Midgut sporozoite count is severely impaired in Alp3KO and Alp5aKO lines. (G) Normalisation of midgut sporozoite count (F) against oocysts per infected midgut (C, D) indicates an impairment of sporozoite formation in Alp5aKO oocysts. Statistics were performed using Mann–Whitney test. ns, not significant; *p < 0.05; **p < 0.01; ****p < 0.0001 (Table S1). For all experiments, at least two biological replicates were performed per parasite line.

### 2.3 Alp5a, but Not Alp3, Is Required for Sporozoite Formation

Hundreds of sporozoites develop per oocyst and egress into the haemocoel to reach and invade the salivary glands. Midgut sporozoites were quantified on days 13–14 and 18–19 post‐infection. For the Alp3KO line, as expected due to very low total oocyst numbers, the quantity of Alp3KO midgut sporozoites was highly reduced compared to the wild‐type control and was rescued by Alp3 complementation (Figure 2F). In case of the Alp5aKO line, total midgut sporozoite numbers were even lower with very few sporozoites observed (Figure 2F). This severe phenotype remained consistent over the course of 5 days and could be rescued by complementation with wild‐type Alp5a. To determine whether the reduction in numbers was simply due to the reduction in total oocysts, sporozoite numbers were divided by the average number of oocysts. This ratio was comparable to wild‐type in case of Alp3KO, indicating that the few oocysts present were able to sporulate efficiently (Figure 2G). However, in the case of the Alp5aKO line, the average number of sporozoites per oocyst was reduced to practically zero (Figure 2G). In summary, Alp3 is not required for sporulation and Alp5a is a critical factor for sporozoite development. Therefore, while both Alps are involved in oocyst formation, Alp5a is critical for oocyst maturation while Alp3 is required for events preceding oocyst formation.

### 2.4 Alp3 Is Required for Ookinete Penetration of the Midgut

In order for an oocyst to form, a *Plasmodium* ookinete needs to develop, penetrate the midgut epithelium, establish itself at the basal lamina and transform into an oocyst. To assess the functional role of Alp3 in these steps, we first investigated whether Alp3KO ookinetes can develop in the mosquito midgut. Fully mature Alp3KO ookinetes were readily observed in in vitro cultures (Figure 1C), and in the blood bolus of infected mosquitoes dissected 24 h after blood meal, indicating no major impact of Alp3 deletion on in vivo ookinete development (Figure 3A). Similar numbers of Alp3KO and wild‐type ookinetes were found in the blood bolus (6000 and 6250 per microliter pelleted blood bolus for Alp3KO and wild‐type, respectively). Next, we investigated whether Alp3KO ookinetes were able to penetrate the midgut epithelium. To this end, *Anopheles* midguts were dissected 24 h post‐infection, washed to remove any ookinetes remaining in the bolus and immuno‐stained using a *Plasmodium*‐specific α‐HSP70 antibody. In the case of the Alp3KO, we detected a more than ten‐fold reduction of parasites in the midgut epithelium (median of 92 and 7 parasites for wild‐type and Alp3KO, respectively) (Figure 3B,C, Figure S4A), indicating a deficiency in epithelial layer penetration. This reduction was apparent regarding ookinetes and transforming ookinetes (tooks) as well as early oocysts (Figure 3D,E, Figure S4B). This reveals that Alp3 plays a critical role in ookinete penetration rather than in early oocyst development. Taken together, our data show that two unique members of the actin superfamily, Alp3 and Alp5a, are critical for parasite progression in mosquitoes and are very likely involved in different processes.

**FIGURE 3:**
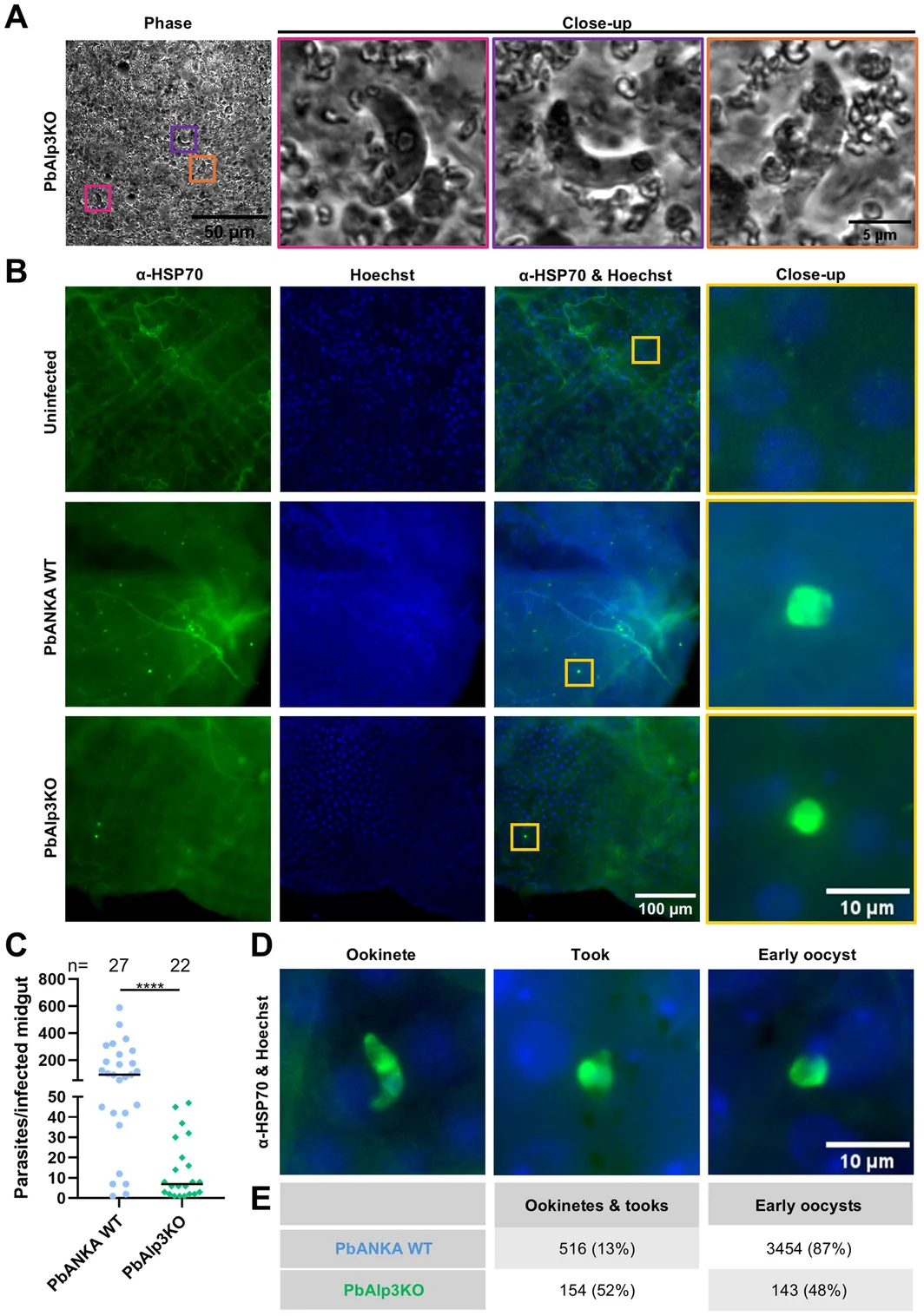
Alp3 is required for midgut epithelium penetration. (A) PbAlp3KO ookinetes were present in blood bolus from mosquitoes dissected 24 h post‐infection. (B) Alp3KO parasites could be identified in the midgut epithelium by immuno‐staining *Anopheles* midguts 24 h post‐infection using a *Plasmodium*‐specific α‐HSP70 antibody. (C) Quantification of parasites from (B) reveals that in the Alp3KO line, parasite numbers in the *Anopheles* midgut 24 h post‐infection are significantly reduced compared to wild‐type. (D) Exemplary images of an ookinete, a transforming ookinete (took) and an early oocyst in the midgut epithelium. (E) Numbers of ookinetes and tooks as well as early oocysts in the midgut were reduced in Alp3KO‐infected midguts compared to wild‐type. Statistics were performed using Mann–Whitney test. **** = *p* < 0.0001 (Table S1). For all experiments, two biological replicates were performed per parasite line.

## 3 Discussion

Actin superfamily members are essential for multiple cellular processes across eukaryotes (Stoddard et al. 2017). *Plasmodium* conventional actin 1 is one of the most highly sequence divergent eukaryotic actins known, with this sequence divergence important for ookinete motility, smooth sporozoite motility and mosquito salivary gland penetration (Douglas et al. 2018; Yee et al. 2022; Kobayashi and Douglas 2025). The Arps subgroup has also been implicated or identified as essential for *Plasmodium* biology. Arp1 is required for efficient parasite blood stage growth (Bushell et al. 2017; Siden‐Kiamos et al. 2010). Arp2 and 3 were originally thought to be absent in apicomplexans but, recently, Alp5b and Alp5a were discovered to be functioning in an atypical Arp2/3 complex that is critical for DNA segregation of activated male gametocytes (Hentzschel et al. 2025). Here, using reverse genetics and microscopy, we identified important roles of Alp3 and Alp5a in *Plasmodium* transmission: Alp3 is required for ookinete penetration of the mosquito midgut epithelium and Alp5a is necessary for oocyst maturation and sporozoite development. Due to the different phenotypes caused at different stages of the parasite life cycle by both KOs, Alp3 and Alp5a seem likely to act independently of each other.

Our knock‐out study identified Alp5a as non‐essential for blood stage growth, male gametogenesis and ookinete motility, yet as a critical factor for ookinete DNA content, oocyst development and subsequent sporozoite formation. Alp5a is expressed in a male gametocyte‐specific manner (Howick et al. 2019) and, in a recent study identifying an atypical Arp2/3 complex in *Plasmodium*, knock‐out of other complex members (ARPC1 and Alp5b) also led to similar effects on DNA content and oocyst development. This further highlights the essential contributions of these Arp2/3 complex partner proteins in the function of the entire atypical complex (Hentzschel et al. 2025) and supports the suggestion that Alp5a is the functional homolog of Arp3. An Alp5a knock‐out line was also recently published and a similar phenotype on oocyst number and size was observed (Varshney et al. 2025). The exact mechanism of Alp5a and the atypical Arp2/3 complex remains unclear. It has been proposed that loss of complex function, and the resulting aneuploidy, could lead to the activation of a post‐meiotic replication checkpoint that arrests the cell cycle in early oocysts and thus represents a delayed death phenotype (Hentzschel et al. 2025). Alternatively, loss of part of the male genome could cause gene dosage effects in early oocysts (Hentzschel et al. 2025) or the loss Alp5a could indirectly impact endomitosis in oocysts (Varshney et al. 2025). Understanding the molecular interactions of this unique actin superfamily member will not only advance our knowledge of the structure–function relationships of Alp5a but also shed light on the molecular mechanisms of efficient DNA segregation in male gametocytes required for oocyst development. Considering the effect of Alp5aKO on DNA content in ookinetes, it is tempting to speculate that there could be a similar effect during sporulation in developing oocysts. Inducible knock‐out methods or promoter swap approaches could be employed in the future to assess whether later life cycle steps require this complex for DNA segregation.

To our knowledge, this is the first in‐depth investigation of the molecular role of Alp3, not only in *Plasmodium*, but in any apicomplexan. We found Alp3 to be important for penetration of the mosquito midgut epithelial layer, a process that to date is still poorly understood (Hentzschel and Frischknecht 2022). Several proteins thus far have been shown to be involved in ookinete penetration of the midgut (Smith and Barillas‐Mury 2016). Similar to the Alp3KO, knock‐out of LIMP did not impair ookinete gliding motility but led to severely reduced oocyst numbers (Egarter et al. 2021). Subtilisin‐like protein PIMMS2 caused reduced midgut epithelium invasion of ookinetes (Ukegbu et al. 2017). CBP‐O, a cyclic nucleotide binding protein in ookinetes, is also required for successful midgut penetration (Kwecka et al. 2025). Further, a quadruple mutant of the micronemal protein Akratin which, unlike the AkratinKO line, was able to exflagellate and form motile ookinetes but these ookinetes failed to penetrate the midgut epithelium (Kehrer et al. 2024). These findings highlight that the very short process of midgut epithelium penetration (Trisnadi and Barillas‐Mury 2020) requires multiple protein contributors. Several phenotypes could account for defective ookinete penetration, including loss of attachment, lack of proper motor engagement while initially penetrating the cell layer and/or a more efficient ejection of penetrating ookinetes by the epithelium. While the molecular mechanisms of Alp3 remain unclear, it is tempting to speculate what the protein's specific role in midgut penetration entails. In a recent study in which the transcriptome of early mosquito stages of the related *P. falciparum* was investigated, Alp3 was annotated as involved in cytoskeleton organisation. Furthermore, it was categorised in a gene cluster concerning rhoptry, nuclear migration and the inner membrane pellicle (Yan et al. 2025). Although we could not detect any defects in gliding motility in Alp3KO ookinetes, penetration of the mosquito midgut epithelium was severely impaired. Interestingly, an analogous observation was made in one of our previous studies on highly divergent *Plasmodium* actin residues in sporozoites, whereby mutant haemolymph sporozoites were generally able to glide to similar extents as wild‐type controls, but were unable to efficiently penetrate salivary glands (Yee et al. 2022). We suggest that the phenotype observed in the Alp3KO could be due to defects in the penetration machinery, possibly caused by slightly modified actin dynamics or reduced secretion of micronemal proteins, whereby sufficient dynamics or micronemal proteins are present to allow for gliding on a uniform glass surface but lack sufficient traction or force in vivo to penetrate the epithelial layer. Indeed, classical Arps have been implicated in regulation of actin dynamics, protein secretion and motility (Chakrabarti et al. 2021; Svitkina 2018) and it thus possible that specialised apicomplexan Arps, including Alp3, could be contributing to one or more of these important steps. Nonetheless, with the current data, we cannot formally exclude the possibility that Alp3 KO ookinetes are more efficiently ejected out of the epithelium during traversal. Tagging strategies, especially when combined with microscopy techniques such as expansion microscopy, could reveal whether Alp3 is associated with the glideosome or micronemes and will shed more light on the specific role Alp3 plays in this step of the parasite life cycle.

Mosquito infection is one of the largest bottlenecks in the *Plasmodium* life cycle. Our work has identified that Alp3 is required for ookinete traversal of the midgut epithelium and Alp5a as critical for oocyst development (Figure 4). These findings highlight the involvement and requirement of unique actin superfamily members that are specialised in penetration of, and *Plasmodium* establishment in, the mosquito midgut.

**FIGURE 4:**
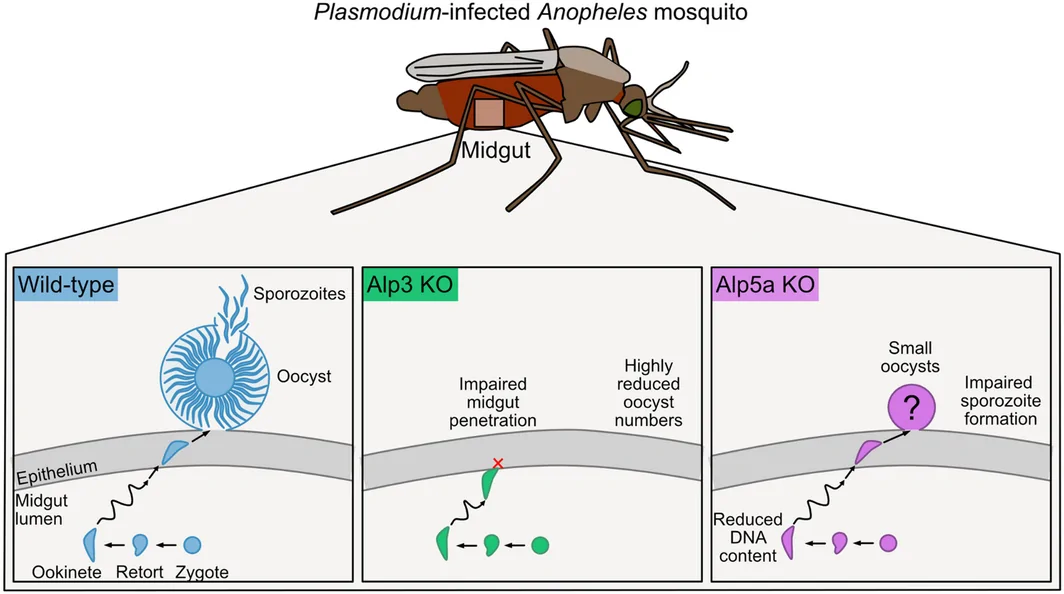
Graphical representation of the roles of Alp3 and Alp5a in *Plasmodium* transmission to mosquitoes. Knock‐out of both Alps leads to strikingly different phenotypes, indicating that they likely function independently of each other.

## 4 Experimental Procedures

### 4.1 In Silico Structure Predictions

In silico structure predictions of *Plasmodium berghei* actin 1 (PBANKA_1459300), Alp3 (PBANKA_0212300) and Alp5a (PBANKA_0811800) were performed using AlphaFold2 (Bryant et al. 2022) and structures were coloured and modelled using open source PyMOL (Schroedinger). DNA and amino acid sequences were obtained from PlasmoDB (Bahl et al. 2003). Amino acid sequence identity and similarity were assessed using the EMBOSS Needle tool (EMBL, Madeira et al. 2024).

### 4.2 Animal Experiments and Mosquito Rearing

All animal work was approved by local authorities (Regierungspraesidium Giessen and Regierungspraesidium Karlsruhe) and performed according to the Federation of European Laboratory Animal Science Associations (FELASA) and Gesellschaft fuer Versuchstierkunde/Society for Laboratory Animal Science (GV‐SOLAS) standard guidelines. Four‐ to six‐week‐old SWISS mice (Janvier Laboratories, France) were used for all animal experiments. *Anopheles stephensi* mosquitoes were reared and maintained according to standard breeding protocols (Benedict and Dotson 2015).

### 4.3 Generation of Knock‐Out and Complementation Parasite Lines

Knock‐out vectors of *Plasmodium berghei* Alp3 (PBANKA_0212300) and Alp5a (PBANKA_0811800) were kindly provided by the PlasmoGEM resource (Schwach et al. 2015). These vectors contain *hDHFR* and *yfcu* genes, both under an eEF1 promotor. Transfection was performed as previously described (Janse et al. 2006) using a Nucleofector (Lonza) and parasites were selected using 0.07 mg/mL pyrimethamine that was added to the drinking water. Correct integration of DNA was investigated by harvesting mouse blood via cardiac puncture, gDNA purification using the DNeasy Blood & Tissue kit (Qiagen) and genotyping PCR (Table S2, Figure S2). Limiting dilution was performed to generate isogenic parasite lines. Asexual growth rates were calculated based on parasitaemia values from the limiting dilution as described before (Klug et al. 2016). To negatively select for parasites without selection cassette, 1.0–1.5 mg/mL 5‐FC was added to the drinking water and limiting dilution was performed. Loss of selection cassette was confirmed via genotyping PCR (Table S2, Figure S3).

For complementation lines, wild‐type Alp genes were obtained from *P. berghei* gDNA (Table S2) and cloned into Pb238 transfection vectors (Douglas et al. 2018) using BssHII and BamHI (NEB). These vectors were linearised using SacII and PmeI (NEB) and transfected as above into negatively selected KO parasites. Limiting dilution was performed and correct integration of the gene was confirmed via genotyping PCR (Table S2).

### 4.4 Exflagellation Assay

Swiss mice were infected with cryo‐preserved *Plasmodium* parasites via intra‐peritoneal injection. When parasitaemia reached 1.5%–2%, blood was harvested via cardiac puncture and blood corresponding to 20 million parasites was transferred into a recipient mouse. Three days later, a drop of tail blood from an infected mouse was placed on a microscopy slide and covered with a coverslip. The slides were incubated at RT in the dark for 9 min. Exflagellation centres were quantified across multiple fields of view under a Zeiss AxioLab A1 light microscope using a 40× objective and phase contrast ring between minutes 9 and 14 post‐activation. Red blood cells were counted in one field. Gametocytaemia was assessed by preparing a Giemsa‐stained blood smear. Exflagellation centres were normalised against gametocyte numbers.

### 4.5 Ookinete Culture

Ookinete culture analysis was performed as described previously (Douglas et al. 2018; Yee et al. 2022). Briefly, mice were infected and fresh blood transfer was performed as described above. Three days later, if one or more exflagellation centres per microscope field could be observed, blood was collected and incubated in ookinete medium (250 mL RPMI1640 + HEPES + glutamine, 12.5 mg hypoxanthine, 2.5 mL penicillin/streptomycin (100×), 0.5 g sodium bicarbonate, 5.12 mg xanthurenic acid (100 μM), 16% (v/v) foetal bovine serum, pH 7.8) at 19°C for 21 h in the dark. Development of ookinetes was confirmed by Giemsa smear of the culture. Ookinetes were enriched by adding a cushion of 10 mL 63% Nycodenz to 10 mL ookinete culture and centrifuging at 1000 rpm/25 min/RT without break. The ookinete‐containing ring at the interface was collected and pelleted at 1000 rpm/10 min/RT with break. The pellet was resuspended in 1 mL ookinete medium.

For motility and speed analysis, 100–200 μL ookinete culture was centrifuged at 13,000 rpm/10 s/RT and 2 μL of the pellet was added on a microscopy slide, covered with a coverslip and sealed with paraffin wax. Ookinetes were imaged under an Axioskop 2 MOT (Zeiss) every 30 s for 15 min using a 40× objective. Movies were analysed using FIJI (Schindelin et al. 2012), employing the manual tracking plug‐in for speed analysis.

For ookinete DNA content analysis, PbAlp3KO and respective wild‐type control ookinetes were fixed in 4% PFA/PBS for 10 min at RT in the dark and washed. In case of PbAlp5aKO and the respective wild‐type control, ookinetes were not fixed. In both cases, ookinetes were stained with 10 μg/mL Hoechst 33342 (Thermo Fisher) in ookinete medium for 10 min at RT in the dark and centrifuged at 13,000 rpm/10 s/RT. The pellet was resuspended in ookinete medium, centrifuged again and 2 μL of the pellet were added to a microscopy slide, covered with a coverslip and sealed with paraffin wax. Ookinetes were imaged under an Axio Observer Z1 (Zeiss) using a 63× objective (DIC channel at 150 ms exposure and Hoechst 33258 channel at 25 ms exposure). Hoechst signal intensity was analysed using a FIJI macro as described previously (Hentzschel et al. 2025).

### 4.6 Mosquito Infection

For infections, a donor mouse was infected with cryo‐preserved parasites and, upon reaching a parasitaemia of 1.5%–2%, blood was harvested. Blood containing 20 million parasites was transferred into two recipient mice. When at least one exflagellation event per field could be detected, the mice were anaesthetised (100 mg/kg ketamine, 3 mg/kg xylazine), placed on a mosquito cage that had been starved for 3–6 h and mosquitoes allowed to feed for 30 min. Mice were sacrificed via cervical dislocation directly after feeding. The mosquito cage was placed in an incubator at 21°C and 70% humidity and mosquitoes were maintained with 10% saccharose with 0.05% PABA and 0.1% NaCl solution.

### 4.7 Characterisation of Mosquito Stage Parasites

Mosquitoes were collected and placed on ice before being briefly dipped into 70% EtOH and transferred to a dish containing PBS. Prepared mosquitoes were then placed on a microscopy slide and midguts dissected under a SMZ1B stereo microscope (Nikon) in PBS. Midguts were collected in PBS on ice and further analyzed.

For mercurochrome staining, midguts were dissected on days 11 and 12 post‐infection and permeabilised with NP‐40 (AppliChem, final concentration 1% in PBS) for 20 min at RT. NP40 solution was removed and midguts were stained in 1 mL 0.1% mercurochrome/PBS for 2 h at RT. The samples were washed 4× with PBS before being aligned on a microscopy slide and covered with a coverslip. Stained midguts were imaged under an Axio Observer Z1 (Zeiss) using a 10× objective with a green filter. Infection rates, oocyst number and oocyst size were analysed using FIJI (Schindelin et al. 2012).

For midgut sporozoite counts, midguts were dissected into 50 μL PBS on days 13, 14, 18 and 19 post‐infection. Dissected midguts were crushed with a pestle for 1 min and diluted with PBS before adding 10 μL of the sample onto a Neubauer counting chamber. Parasites were counted on a light microscope with phase contrast ring using a 40× objective.

For in vivo ookinete and early oocyst analysis, midguts of female *Anopheles* mosquitoes were dissected 24 h post‐infection and carefully sliced open longitudinally to remove the blood bolus. To analyse ookinete formation in the blood bolus, blood boluses of 15 infected midguts were collected and fixed with 4% PFA/PBS for 1 h at RT and washed 4× in PBS. For detection of ookinetes, the samples were pelleted (7000 rpm/2 min/RT) and supernatant removed. 2 μL of the wet pellet was placed on a microscopy slide, covered with a coverslip and sealed with paraffin wax before imaging on a DM6 B microscope (Leica) using a 40× objective. Images were analysed using FIJI (Schindelin et al. 2012). To quantify ookinetes in the blood bolus, fixed blood bolus samples were pelleted by centrifugation and the supernatant was removed. 1 μL of the pellet was collected and added to 99 μL PBS. After thorough mixing, 10 μL of the sample were pipetted onto a Neubauer counting chamber. The number of ookinetes was assessed under a BX50 microscope (Olympus) with DIC filter using a 40× objective.

For ookinete analysis in the midgut epithelium, empty midguts were fixed with 4% PFA/PBS for 1 h at RT. Fixed midguts were washed 4× with PBS and permeabilised with 0.1% Triton‐X‐100/PBS for 10 min at RT. The samples were washed, blocked with 5% BSA/PBS for 1 h at RT and stained overnight with a *Plasmodium*‐specific α‐HSP70 antibody. After another wash step, parasites were visualised using an α‐rabbit antibody coupled with AlexaFluor 488 (Invitrogen) for 1 h. After 30 min, Hoechst 33342 was added (Thermo Fisher, final concentration 1 μg/μL). The samples were washed again, and the midguts were gently placed on a microscopy slide, covered with a coverslip and sealed around the edges using paraffin wax. Imaging was performed using a DM6 B fluorescence microscope (Leica) using a 40× objective and an Axio Observer Z1 with an Apotome (Zeiss) using a 63× objective. Image analysis was performed using FIJI (Schindelin et al. 2012).

## Author Contributions

**Caroline Busse:** investigation, writing – original draft, methodology, visualization, writing – review and editing, formal analysis. **Andrea Diers:** investigation, methodology. **Ross G. Douglas:** conceptualization, funding acquisition, writing – original draft, writing – review and editing, visualization, project administration, supervision. **Friedrich Frischknecht:** conceptualization, writing – review and editing, project administration, supervision. **Annika M. Binder:** investigation, methodology. **Yukino Kobayashi:** investigation, methodology.

## Funding

This work was supported by Deutsche Forschungsgemeinschaft, DO 2329/3‐1.

## Ethics Statement

All animal work was approved by local authorities (Regierungspraesidium Giessen and Regierungspraesidium Karlsruhe) and performed according to the Federation of European Laboratory Animal Science Associations (FELASA) and Gesellschaft fuer Versuchstierkunde/Society for Laboratory Animal Science (GV‐SOLAS) standard guidelines.

## Conflicts of Interest

The authors declare no conflicts of interest.

## Supporting information


**Figure S1:** Alp3 and Alp5a are highly sequence divergent from actin and are predicted to have an actin fold. (A) AlphaFold2 structure prediction of *P. berghei* actin 1, Alp3 and Alp5a. Gray outlines represent four subdomains which, in addition to a nucleotide binding cleft in the centre, are characteristic for the actin‐fold. Alp3 and Alp5a share the conserved actin‐fold core but have large peripheral insertions. (B), (C) Amino acid sequence identity [%] (B) and similarity [%] (C) are low between actin 1 and Alp3 or Alp5a from *P. berghei*.
**Figure S2:** Genotyping of *P. berghei* Alp3 and Alp5a knock‐out lines. (A) Genotyping strategy of Alp3 knock‐out line. (B) Genotyping gel shows correct integration of the knock‐out vector. (C) Genotyping strategy of Alp5a knock‐out line. (D) Genotyping gel shows correct integration of the knock‐out vector. Chr, chromosome; DHFR, dihydrofolate reductase; eEF1α, eucaryotic translation elongation factor 1α; HA, haemagglutinin; hom, homology region; kb, kilo base pairs; KO, knock‐out; ORF, open reading frame; WL, whole locus; yfcu, yeast cytosine deaminase and uridyl‐phosphoribosyltransferase.
**Figure S3:** Genotyping of *P. berghei* Alp3 and Alp5a complementation lines. (A) Genotyping strategy of Alp3 complementation line. (B) Genotyping gel shows correct integration of the complementation vector. (C) Genotyping strategy of Alp5a complementation line. (D) Genotyping gel shows correct integration of the complementation vector. Chr, chromosome; comp., complementation; DHFR, dihydrofolate reductase; eEF1α, eucaryotic translation elongation factor 1α; HA, haemagglutinin; hom, homology region; kb, kilo base pairs; KO, knock‐out; ORF, open reading frame; WL, whole locus; yfcu, yeast cytosine deaminase and uridyl‐phosphoribosyltransferase.
**Figure S4:** Additional examples of fixed and stained *Plasmodium*‐infected *Anopheles* midguts. (A) Both wild‐type and Alp3KO parasites could be detected in the midgut epithelium. Parasites were immuno‐stained using an anti‐HSP70 antibody and imaged with a fluorescence microscope using an Apotome (Zeiss). Two biological replicates were performed per parasite line. (B) Representative images of an ookinete and an early oocyst in the midgut epithelium.
**Table S1:** Statistical test results summary.
**Table S2:** Primers used for cloning and genotyping PCRs.

## Data Availability

The data that support the findings of this study are available from the corresponding author upon reasonable request.
