# Supplementary material for: Plasmodium Actin‐Like Proteins 3 and 5a Are Required for Subsequent Steps of Mosquito Infection
Source: Mol Microbiol. 2026 Aug 21;126(4):451–61. doi: 10.1111/mmi.70108 (PMC13644374; doi:10.1111/mmi.70108)
Supplement: Supplementary file 1 [file MMI-126-451-s001.pdf]

Supporting Information

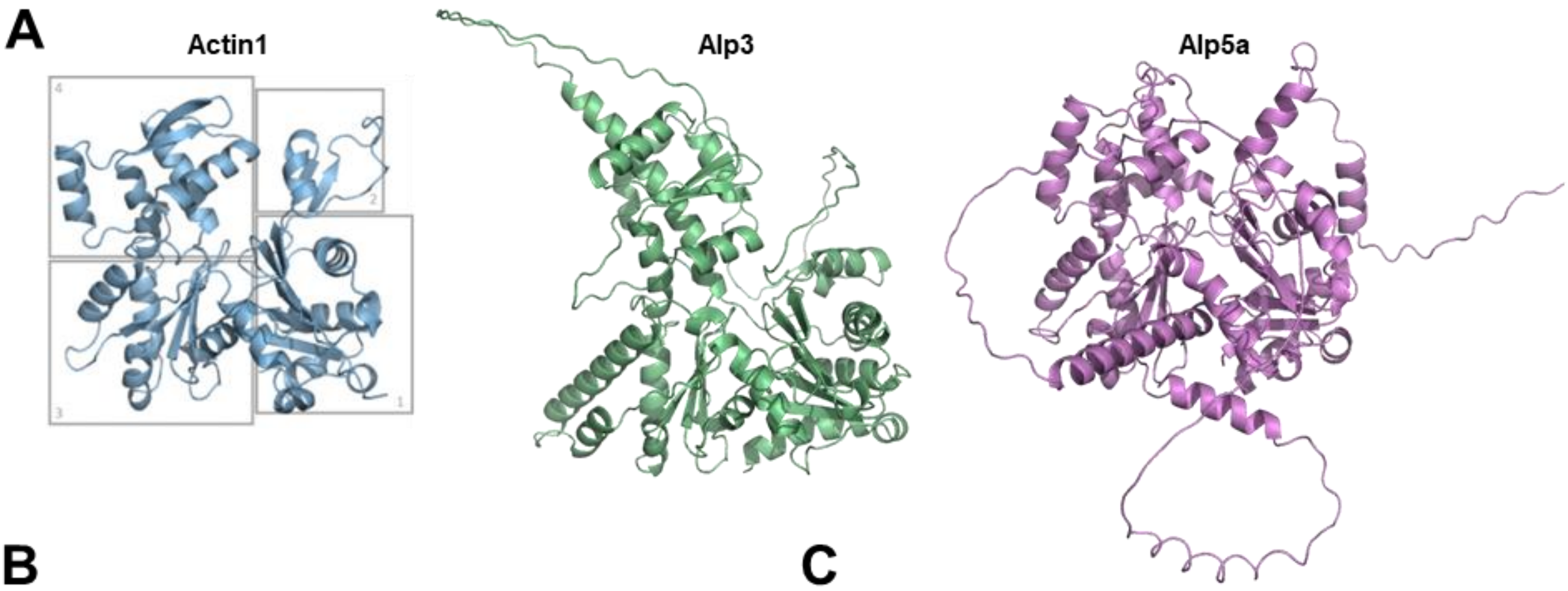

B

Identity	Actin1	Alp3	Alp5a
Actin1	100	13	14
Alp3	13	100	18
Alp5a	14	18	100

C

Similarity	Actin1	Alp3	Alp5a
Actin1	100	28	25
Alp3	28	100	30
Alp5a	25	30	100

Figure S1: Alp3 and Alp5a are highly sequence divergent from actin and are predicted to have an actin fold. (A) AlphaFold2 structure prediction of *P. berghei* actin 1, Alp3 and Alp5a. Gray outlines represent four subdomains which, in addition to a nucleotide binding cleft in the centre, are characteristic for the actin-fold. Alp3 and Alp5a share the conserved actin-fold core but have large peripheral insertions. (B), (C) Amino acid sequence identity [%] (B) and similarity [%] (C) are low between actin 1 and Alp3 or Alp5a from *P. berghei.*

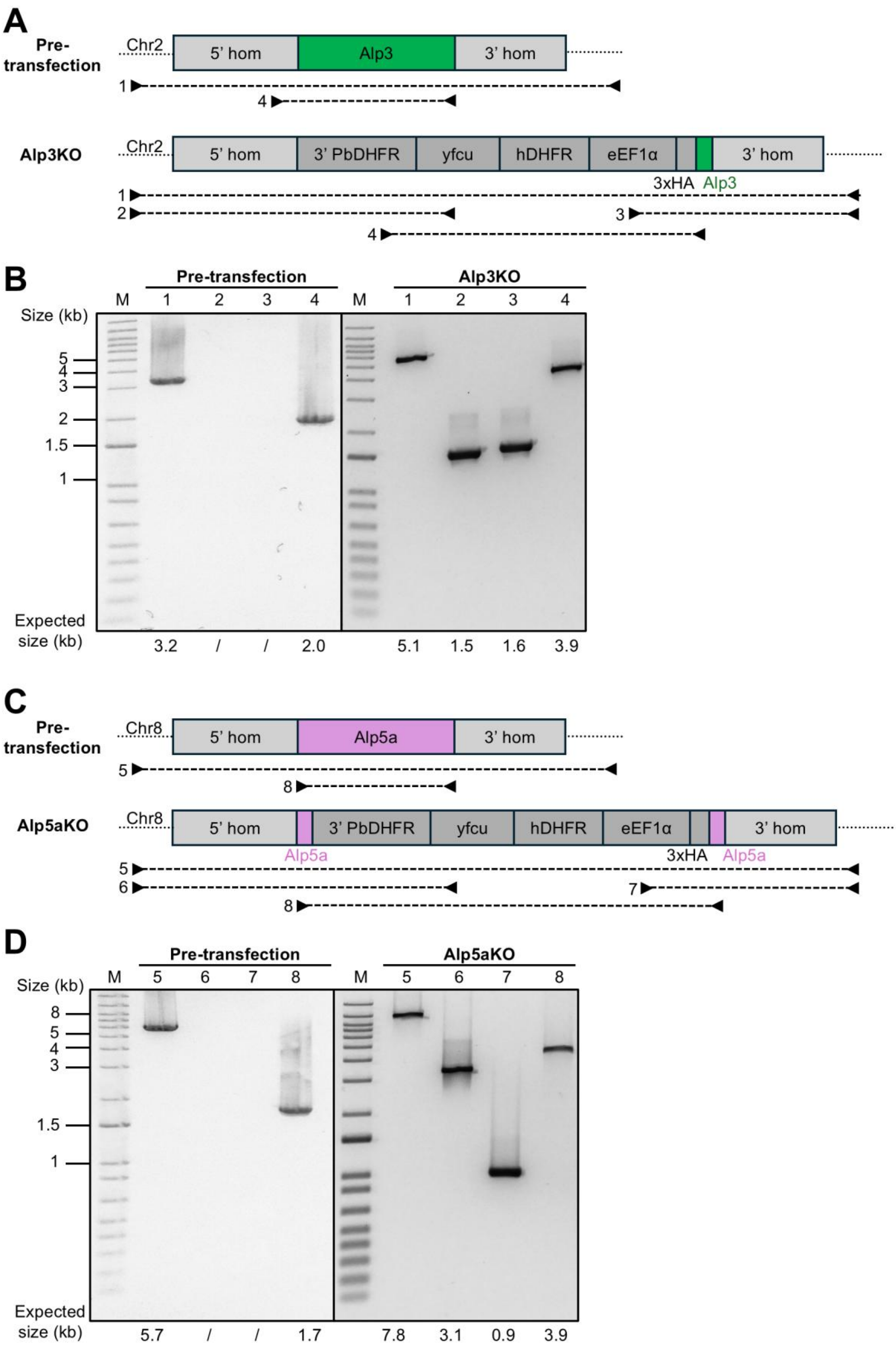

Figure S2: Genotyping of *P. berghei* Alp3 and Alp5a knock-out lines. (A) Genotyping strategy of Alp3 knock-out line. (B) Genotyping gel shows correct integration of the knock-out vector. (C) Genotyping strategy of Alp5a knock-out line. (D) Genotyping gel

shows correct integration of the knock-out vector. Chr, chromosome; DHFR, dihydrofolate reductase; eEF1α, eucaryotic translation elongation factor 1α; HA, haemagglutinin; hom, homology region; kb, kilo base pairs; KO, knock-out, ORF, open reading frame; WL, whole locus, yfcu, yeast cytosine deaminase and uridyl-phosphoribosyltransferase.

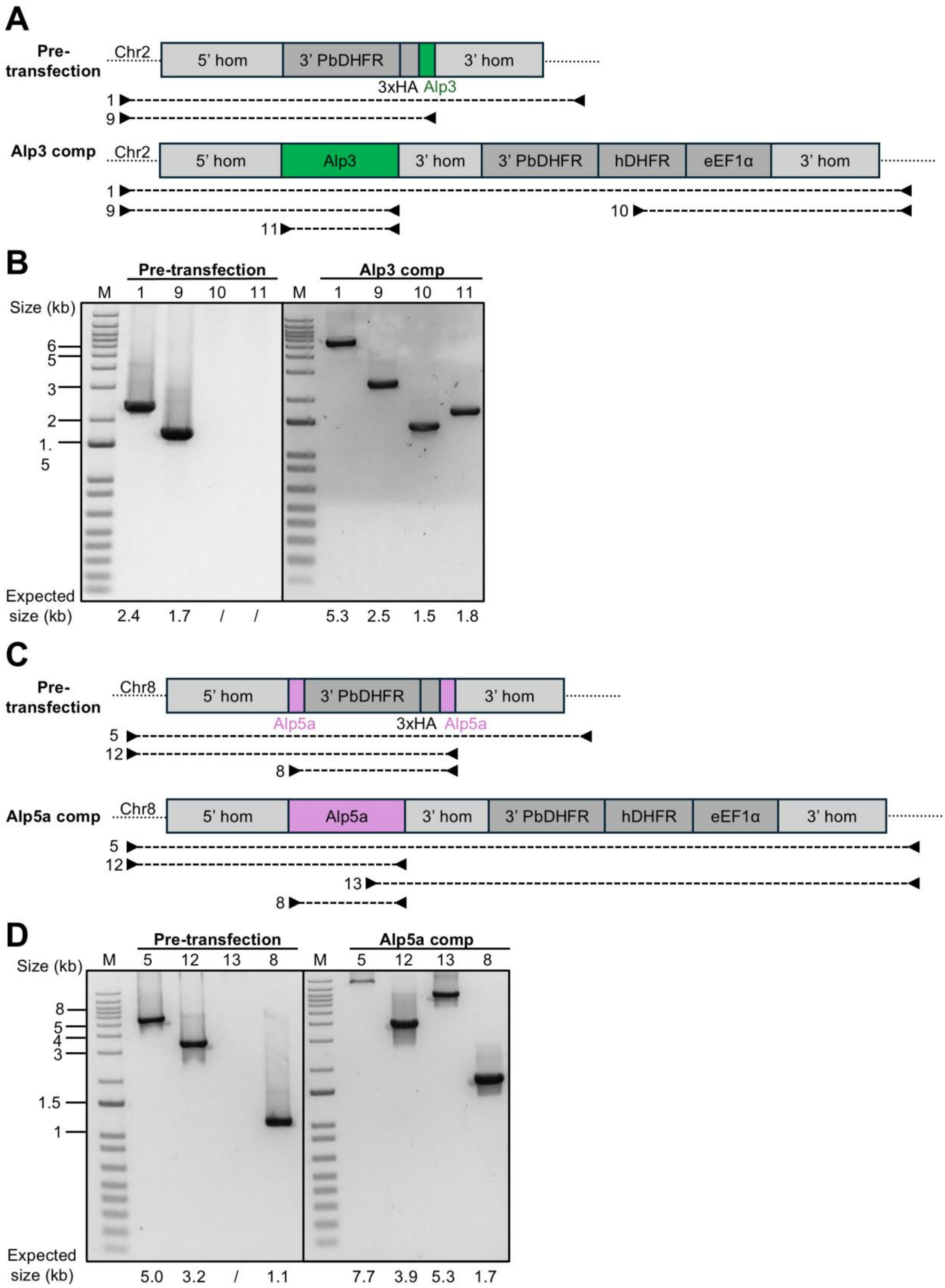

Figure S3: Genotyping of *P. berghei* Alp3 and Alp5a complementation lines. (A) Genotyping strategy of Alp3 complementation line. (B) Genotyping gel shows correct integration of the complementation vector. (C) Genotyping strategy of Alp5a complementation line. (D) Genotyping gel shows correct integration of the complementation vector. Chr, chromosome; comp, complementation; DHFR,

dihydrofolate reductase; eEF1α, eucaryotic translation elongation factor 1α; HA, haemagglutinin; hom, homology region; kb, kilo base pairs; KO, knock-out, ORF, open reading frame; WL, whole locus; yfcu, yeast cytosine deaminase and uridyl-phosphoribosyltransferase.

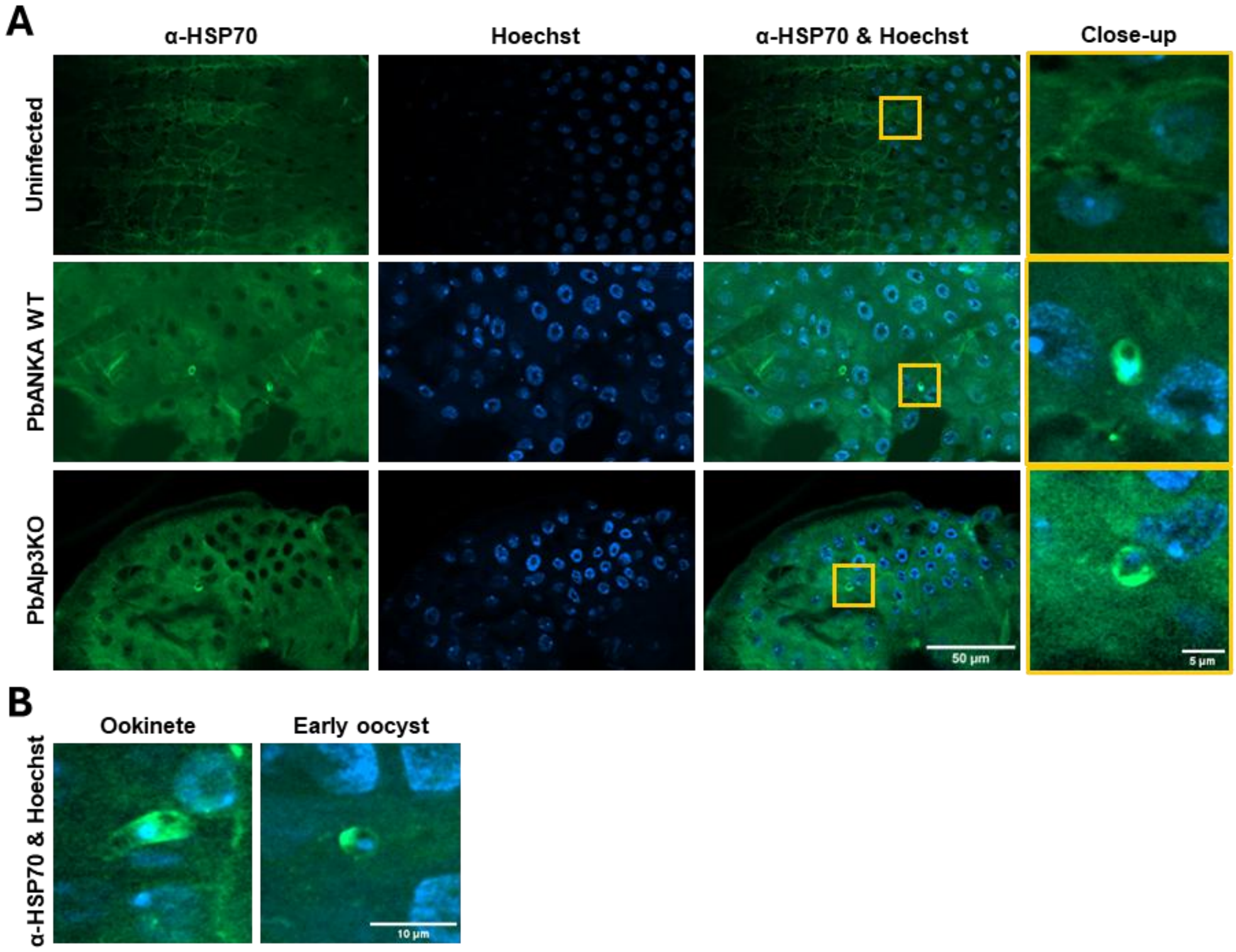

Figure S4: Additional examples of fixed and stained *Plasmodium*-infected *Anopheles* midguts. (A) Both wild-type and Alp3KO parasites could be detected in the midgut epithelium. Parasites were immuno-stained using an anti-HSP70 antibody and imaged with a fluorescence microscope using an Apotome (Zeiss). Two biological replicates were performed per parasite line. (B) Representative images of an ookinete and an early oocyst in the midgut epithelium.

Table S1: Statistical test results summary.

Experiment	Figure	Compared parasite lines	P-value	Significant
Exflagellation assay	1b	Alp3KO vs. wild-type	0.6810	No
		Alp5aKO vs. wild-type	0.8215	No
Ookinete motility	1d	Alp3KO vs. wild-type	0.4739	No
	1f	Alp5aKO vs. wild-type	0.7742	No
Ookinete speed	1e	Alp3KO vs. wild-type	0.3311	No
	1g	Alp5aKO vs. wild-type	0.5857	No
Ookinete Hoechst signal intensity	1i	Alp5aKO vs. wild-type	<0.0001	Yes
	1j	Alp3KO vs. wild-type	0.0007	Yes
Midgut infection rates	2c	Alp3KO vs. wild-type	0.4286	No
		Alp3 complementation vs. wild-type	>0.9999	No
		Alp5aKO vs. wild-type	0.5476	No
		Alp5a complementation vs. wild-type	0.2857	No
Oocyst numbers	2d	Alp3KO vs. wild-type	<0.0001	Yes
		Alp3 complementation vs. wild-type	0.1966	No
		Alp5aKO vs. wild-type	<0.0001	Yes
		Alp5a complementation vs. wild-type	0.0106	Yes
Oocyst size	2e	Alp3KO vs. wild-type	0.0173	Yes
		Alp3 complementation vs. wild-type	0.0018	Yes
		Alp5aKO vs. wild-type	<0.0001	Yes
		Alp5a complementation vs. wild-type	<0.0001	Yes
Midgut sporozoites/mosquito day 13/14	2f	Alp3KO vs. wild-type	0.0040	Yes
		Alp3 complementation vs. wild-type	0.9677	No
		Alp5aKO vs. wild-type	0.0007	Yes
		Alp5a complementation vs. wild-type	0.7111	No
Midgut sporozoites/mosquito day 18/19	2f	Alp3KO vs. wild-type	0.0040	Yes
		Alp3 complementation vs. wild-type	0.0848	No
		Alp5aKO vs. wild-type	0.0016	Yes
		Alp5a complementation vs. wild-type	0.4606	No
Parasites per infected midguts	3c	Alp3KO vs. wild-type	<0.0001	Yes

Table S2: Primers used for cloning and genotyping PCRs.

Gene	Direction	Primer sequence (5' - 3')
Alp3	Forward	ATCACTTTTTCTTTTCTTGATTTGGC
	Reverse	CAGAAATGATCTTGACGACG
Alp5a	Forward	TGTGTCTATGCTTTTCTATCATGC
	Reverse	AAGACCGTTGTTTTTATTGCG

Primer pair	Direction	Primer sequence (5' - 3')
1	Forward	CTTGATTTGGCATAATACTATGAG
	Reverse	GCAGAAATGATCTTGACGACG
2	Forward	CTTGATTTGGCATAATACTATGAG
	Reverse	GAGAGGTGTTAAGCCAGAG
3	Forward	GGGGTGAGCATTTAAAGC
	Reverse	GCAGAAATGATCTTGACGACG
4	Forward	GTTATGTTTAACAATATACACGC
	Reverse	ATATCTCGAGCTATCTTTTAAATGTCATGTAGACA
5	Forward	CATTCCTTTGTACAGCAACAG
	Reverse	TATGTCACAACATCCTACTCC
6	Forward	CATTCCTTTGTACAGCAACAG
	Reverse	GAGAGGTGTTAAGCCAGAG
7	Forward	GGGGTGAGCATTTAAAGC
	Reverse	ATATCTCGAGTTAATACAATAACTTTCCTTGTAAG
8	Forward	ATATGCTAGCAGCATGAATAAAAAGGACGAAATA
	Reverse	ATATCTCGAGTTAATACAATAACTTTCCTTGTAAG
9	Forward	CTTGATTTGGCATAATACTATGAG
	Reverse	ATATCTCGAGCTATCTTTTAAATGTCATGTAGACA
10	Forward	GTGGAGGTTCCTTGAGTTC
	Reverse	GCAGAAATGATCTTGACGACG
11	Forward	ATATGCTAGCGAAATAAAATCCAAAAAATATGATAAC
	Reverse	ATATCTCGAGCTATCTTTTAAATGTCATGTAGACA
12	Forward	CATTCCTTTGTACAGCAACAG
	Reverse	ATATCTCGAGTTAATACAATAACTTTCCTTGTAAG
13	Forward	CGAAACATGGAGAGAATTAGG
	Reverse	TATGTCACAACATCCTACTCC
